# Relapsing Episodes of Loss of Consciousness in a Patient With Hepatocellular Carcinoma

**DOI:** 10.14740/wjon790w

**Published:** 2014-12-03

**Authors:** Anastasios Vagionas, Stelios Tigas, Panagiotis Oikonomou, George Pentheroudakis, Vassiliki Malamou-Mitsi, Nicholas Pavlidis

**Affiliations:** a Department of Medical Oncology, University of Ioannina, Greece; b Department of Endocrinology, University Hospital of Ioannina, Greece; c Department of Pathology-Cytology, University of Ioannina, Greece

**Keywords:** Hepatocellular carcinoma, Loss of consciousness, Insulin-like growth factor, Hypoglycemia

## Abstract

Hypoglycemia is common in people with diabetes treated with insulin or oral medications such as sulphonylureas or other secretagogues, and constitutes a relatively rare paraneoplastic syndrome in patients with a variety of mesenchymal or epithelial tumors. In this case report we present a 51-year-old patient with metastatic hepatocellular carcinoma and persistent, severe, symptomatic hypoglycemia and we discuss management options and review the relevant medical literature.

## Introduction

Hypoglycemia accompanied by impaired level of consciousness is a common medical emergency, often a side effect of antidiabetic treatment. Tumor-related hypoglycemia is less common and may be a feature of either insulinomas or of a variety of non-islet cell tumors [1, 2] (Table 1) [3-7].

**Table 1:** Non-islet Cell Tumors Associated With Hypoglycemia

Tumor	% of total
Tumors of mesenchymal origin	41
Mesothelioma	8
Hemangiopericytoma	7
Solitary fibrous tumor	7
Leiomyosarcoma/gastrointestinal stromal tumor	6
Fibrosarcoma	5
Others	8
Tumors of epithelial origin	43
Hepatocellular	16
Stomach	8
Lung	4
Colon	4
Pancreas (non-islet cell)	3
Prostate	2
Adrenal	2
Undifferentiated	2
Kidney	1
Others	1
Tumors of neuroendocrine origin	1
Tumors of hematopoietic origin	1
Tumors of unknown origin	14

Data extracted from Zapf (1993), Frystyk et al (1998), Marks and Teale (1998), Fukuda et al (2006) and Tsuro et al (2006) [3-7].

Hypoglycemia is an uncommon symptom in hepatocellular carcinoma (HCC) [8]. In this report, we present the case of a patient with HCC and hypoglycemia-related loss of consciousness, briefly review the relevant literature and discuss the differential diagnosis, diagnostic workup and management strategy.

## Case Report

**Figure 1:**
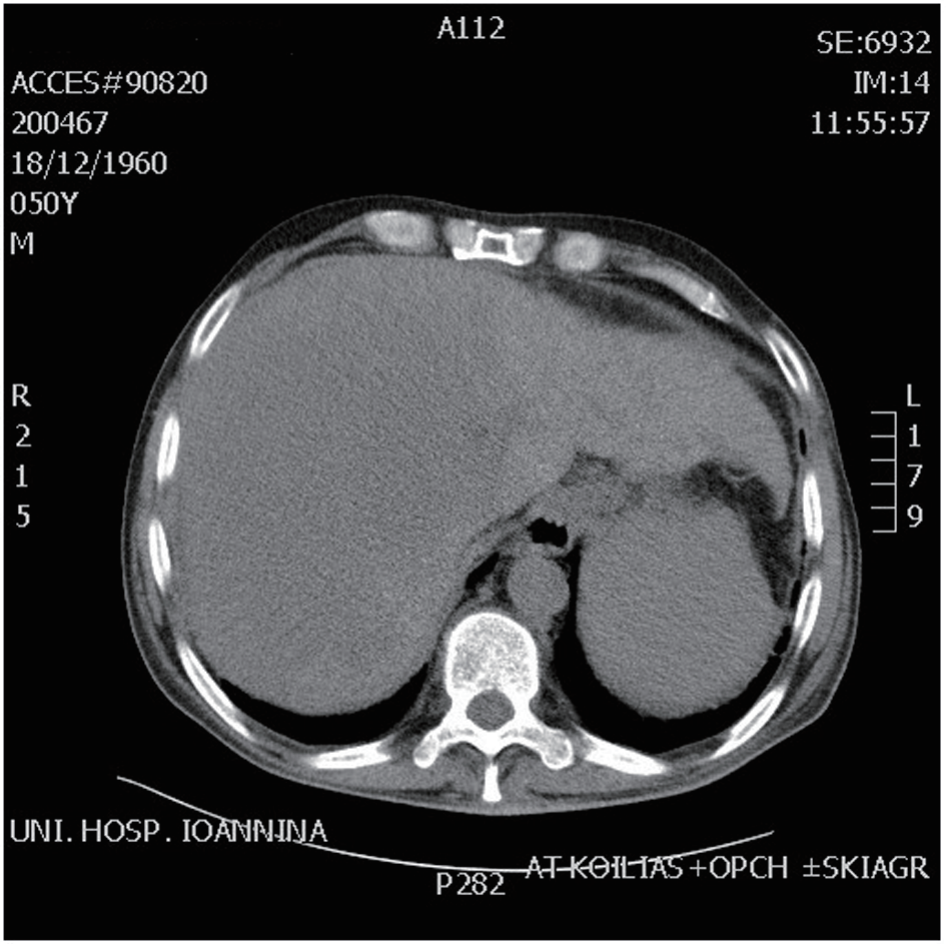
CT scan abdomen showing huge mass in the right lobe of liver.

**Figure 2:**
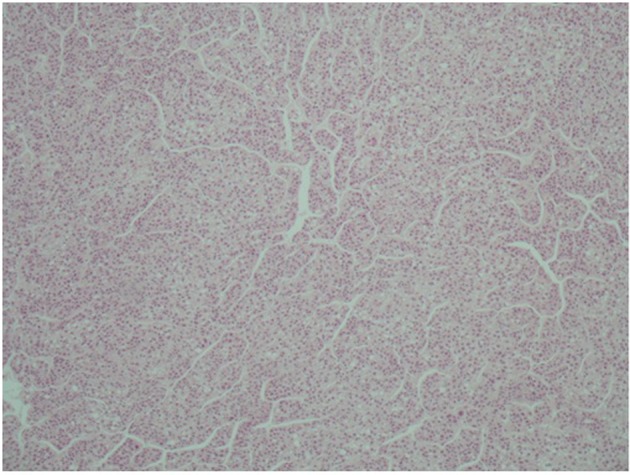
Microscopic appearance of hepatocellular carcinoma. Note the trabecular pattern of growth (H&E, × 10).

**Figure 3:**
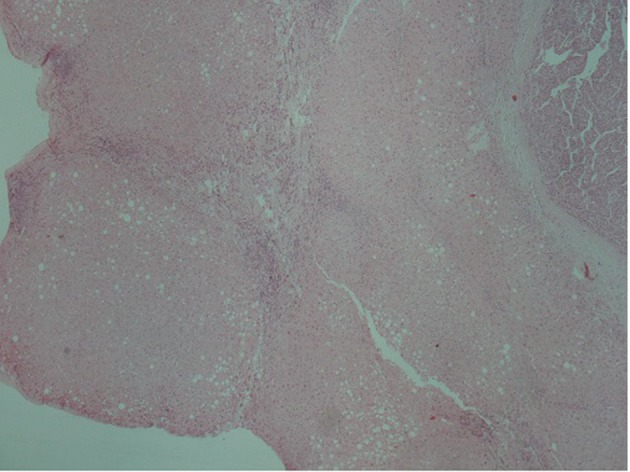
Hepatocellular carcinoma (arrow). In the adjacent liver parenchyma incomplete cirrhotic nodules (star) and fatty changes were observed (H&E, × 4).

A 51-year-old patient with a history of active hepatitis-B viral infection, psoriatic arthritis, heavy alcohol and tobacco use, was referred to the Oncology Department having been recently diagnosed with metastatic HCC (stage IVB). A sizeable mass had been identified in the right liver lobe (Fig. 1) and bone metastases in the thoracic and lumbar vertebrae. The diagnosis was established by liver biopsy (Fig. 2) and a significantly elevated serum alpha-fetoprotein level (above 2,500 ng/mL reference values below 8 ng/mL). The biopsy was consistent with a poorly differentiated HCC and showed evidence of micronodular cirrhosis (Fig. 3). The patient was palliatively treated with sorafenib (multikinase inhibitor) 400 mg po bid.

The patient had been experiencing recurring episodes of loss of consciousness over the previous 3 months. Following one of these episodes, which was preceded by dizziness and profuse sweating, he was admitted to the oncology ward for further management. He reported no use of illicit drugs or antidiabetic medications, no fever or melena.

During the episode, plasma glucose was low at 30 mg/dL and the patient recovered fully following the administration of intravenous (IV) glucose solution (50 mL of dextrose 50%). Physical examination revealed hepatomegaly 3 cm below the right costal margin, diffuse psoriatic rash and palm spider nevi. His blood pressure, heart rate and electrocardiogram were normal. Neurological examination did not reveal any focal deficits. Apart from low plasma glucose concentration, laboratory testing (full blood counts, serum chemistry, coagulation panel, and toxicologic screening) did not specify any other pathological findings (Table 2).

**Table 2:** Laboratory Tests

Laboratory test	Results	Units	Reference values
WBC	7.420	10^3^/µL	7.000 - 11.000
HG	14.40	g/dL	12 - 18
Ht	42.3	%	36 - 48
PLT	222	10^3^/µL	150.000 - 400.000
PT	13.6	s	Normal
INR	1.03	-	0.8 - 1.2
Glu	30	mg/dL	70 - 125
Ur	18	mg/dL	11 - 54
Cr	0.6	mg/dL	0.6 - 1.2
BILIRUBIL	0.6	mg/dL	0.1 - 1
BILL-DIRECT	0.16	mg/dL	0.01 - 0.2
AST	91	IU/L	10 - 35
ALT	42	IU/L	10 - 35
γGT	233	IU/L	10 - 52
ALP	97	IU/L	Adults 30 - 125 IU/L
Total protein	6.7	g/dL	6 - 8.4
Albumin	2.8	g/dL	3.4 - 5
LDH	392	U/L	115 - 230
Calcium	8.8	mg/dL	8.2 - 10.6
Sodium	144	mEq/L	135 - 153
Potassium	3.23	mEq/L	3.5 - 5.3

The patient was classified having Child-Pugh A stage liver cirrhosis. The Child-Pugh score (sometimes the Child-Turcotte-Pugh score) is used to assess the prognosis of chronic liver disease, mainly cirrhosis. Although it was originally used to predict mortality during surgery, it is now used to determine the prognosis, as well as the required strength of treatment and the necessity of liver transplantation. The score employs three laboratory measures (total bilirubin, serum albumin, PT or INR) and two clinical measures (ascites and hepatic encephalopathy). Each measure is scored 1 - 3, with 3 indicating most severe derangement. Over the first week of his hospitalization, he went on to develop episodes of loss of consciousness with documented hypoglycemia on a daily basis, some of which were accompanied by seizures. Endocrinologic consultation was sought and further diagnostic workup was carried out. During hypoglycemia, his serum insulin levels were appropriately low (glucose 10 mg/dL, range 70 - 125 and insulin levels 0.5 µIU/mL, range 1.9 - 23.0) excluding insulinoma. Furthermore, his serum cortisol level during hypoglycemia was adequate (26.0 µg/dL, reference values 6.7 - 22.6) and the serum thyroid stimulating hormone and free T4 were within the normal range at 1.56 µIU/mL and 1.03 with reference values 0.34 - 5.6 and 0.6 - 1.37 respectively) excluding adrenocortical insufficiency and hypothyroidism as a cause of the hypoglycemic episodes. Levels of C-peptid and GABA were not measured in this case due to non-availability of this test, but insulin-like growth factor (IGF-I) was determined by enzyme-linked immunosorbent assay and was found 29.4 ng/mL, while its normal adjusted age-specific levels range between 51 and 297 ng/mL (NR: 50 - 297 ng/mL for age 51 - 60 years). The level of IGF-II, measured by radioimmunoassay after acid-alcohol extraction (test performed by Esoterix Endocrinology) was 485 ng/mL (adults range 288 - 736). In addition, a glucagon stimulation test was performed as follows: during an episode of spontaneous hypoglycemia, glucagon (1 mg, 1 mg/mL solution) was administered as a bolus IV injection. Blood was collected at -10, 0, 15, 30 and 45 min and checked for serum glucose and insulin levels (Table 3). During the test the levels of insulin were suppressed and the glucose levels were increased by > 30 mg/dL, given the suggestion that the patient responded to glucagon administration at the beginning, supporting the hypothesis that the hypoglycemia was due to the paraneoplastic secretion of a factor with insulin-like action. All patients with insulinoma responded to glucagon with a rise in blood serum glucose levels, indicating adequate glycogen stores. The patients with non-islet cell tumor hypoglycemia (NICTH) had mixed responses suggesting that hypoglycemia was due to an insulin-like tumor product. In addition, if the levels of glucose did not rise, it was indicated that hypoglycemia was due to poor hepatic glycogen reserve/liver failure but our patient had an adequate liver remnant at the moment. Finally, we decided to have an immunohistological evidence of IGF-I and IGF-II factors of the primary tumor but could not perform due to non-availability of the test and financial constraints (Table 4). The low insulin and IGF-I, normal IGF-II levels, IGF-II/IGF-I ratio, together with the results of the glucagon stimulation test were suggestive of non-islet-cell tumor hypoglycemia.

**Table 3:** Glucagon Stimulation Test

Time (min)	Glucose (NR: 70 - 125 mg/mL)	Insulin (NR: 1.9 - 23 µIU/mL)
10 before	30	0.6
0	89	0.7
15	155	1.1
30	146	0.6
45	141	0.5

NR: normal ratio.

**Table 4:** Workup of Hypoglycemia

Modality	Results	Conclusion
Serum glucose levels	30 mg/dL (70 - 125)	Hypoglycemia
Clinical manifestations	Hepatomegaly, palmar erythema spider nevi	Hepatic disease
Complete blood count	Normal	-
Serum chemistry	Normal	Child Pugh A
Coagulation test	Normal	Child Pugh A
AFP	7.794 ng/mL (<9 ng/mL)	MCC
TSH	1.56 µIU/mL (0.34 - 5.6)	Normal, exclusion of hypothyroidism
Serum cortisol	26 mg/dL (6.7 - 22.6)	Adequate response, exclusion of adrenocortical insufficiency
Insulin	0.5 µIU/mL (1.9 - 23 µIU/mL)	Suppressed levels during hypoglycemia (excludes insulinoma)
Glucagon test	Positive	Paraneoplastic insulin-like secretion
IGF-I	29.4 ng/mL (51 - 297)	Low
IGF-II	485 ng/mL (288 - 736)	Normal
IGF-II/IGF-I ratio	> 10:1	Consistent with non-islet cell tumor hypoglycemia
Immunohistological stains of IGF-II	Not defined	-
CT scan abdomen	Positive	Large tumor volume

The patient received food-additives rich in carbohydrates, oral corticosteroids (dexamethasone 4 mg three times daily) and diazoxide (3 - 8 mg/kg/day orally divided in three doses therapy not really indicated as it works by reducing insulin secretion) with no apparent effect on control of hypoglycemia, before embarking on glucagon. He received IV glucagon at initial dose 0.06 mg/h, followed by 0.30 mg/h by continuous pump infusion, supplemented by carbohydrate-rich nutrition. Despite an initial good response lasting for 7 days, hypoglycemia episodes reappeared. A new abdominal CT scan highlighted the HCC lesion occupying the largest area of the right lobe (Fig. 1). Regional intra-arterial chemotherapy via the hepatic artery was administered by interventional radiology in the form of 700 mg fluorouracil and 700 mg gemcitabine together diluted with 100 cc normal saline, aiming for cytoreduction of IGF-producing malignant clone. Neither this method induced the desired effect. The patient’s clinical and laboratory deterioration was gradual (Child-Pugh C stage) over the third to sixth weeks of his hospitalization, resulting in coma and death on day 58 due to esophageal varices bleeding.

## Discussion

Primary tumors of the liver represent the sixth most common malignancy worldwide, and the third most common cause of death from cancer. The global incidence of the disease accounts for approximately 626,000 new cases annually, with a male/female ratio of about 2.4:1 [9]. In general, these tumors have a poor prognosis, compounded by background liver disease in the majority of patients. The clinical manifestations of advanced HCC are miscellaneous including decompensated cirrhosis, tumoral symptoms (abdominal pain, weight loss, anorexia, weakness and fullness), fever, metastasis, and paraneoplastic syndromes. Occasionally, central necrosis or acute hemorrhage into the peritoneal cavity leads to death.

Paraneoplastic syndromes in HCC are not uncommon [10]. A variety have been described. The most important ones include hypoglycemia (also caused by end-stage liver failure), ranging from 4% to 27% [8]. Other paraneoplasmatic syndromes associated with hepatoma include erythrocytosis, hypercalcemia, hypercholesterolemia, dysfibrinogenemia, carcinoid syndrome, increased thyroxin-binding globulin, sexual changes and porphyria cutanea tarda [11, 12].

Two types of hypoglycemia in HCC have been described by Mc Fadzean et al [13]. Type A is a mild to moderate severity hypoglycemia that occurs in terminal stages, especially in rapidly growing large tumors. When the less common type B occurs in 5% of cases early in the course of the disease, it suggests a paraneoplastic manifestation. In the present patient, severe hypoglycemia occurred in the late stage of the disease. The occurrence of hypoglycemia in type A has either no hypoglycemia or developing hypoglycemia only few weeks before their death. In these patients, the tumor was rapidly growing and poorly differentiated and there was rapid wasting and profound muscle weakness. It was concluded that in type A patients, hypoglycemia occurs solely as a consequence of a progressive increase in demand for glucose by the tumor coupled with a progressive reduction in glucose supply, in part due to progressive encroachment on the residual liver tissue by the tumor and also to undernutrition. These consist some of the differences between the two types of hypoglycemia.

The most common cause of chronic hypoglycemia in cancer patients, is an insulin-secreting islet-cell tumor. The next most common cause is a tumor producing IGF-II [14]. Also, liver failure and increased glucose consumption by the tumor may be additional factors, contributing to the development of hypoglycemia. In NICTH, IGF-II mRNA overexpressed in tumor cells results in the synthesis of incompletely processed IGF-II (Big IGF-II). Normally, most of the IGFs form a 150 kDa ternary complex with IGF binding proteins; however, Big IGF-II primarily forms a 40 - 50 kDa binary complex with IGF binding proteins. The binary complexes have a higher biological activity to interact with insulin receptors in the liver, adipocytes and muscles, leading to the inhibition of glyconeogenesis and more glucose uptake by skeletal muscle [15]. The binary complexes also have a greater capillary permeability and thus cause a strong insulin-like effect through the insulin receptors, profound hypoglycemia thereupon developed [2, 16]. When the incompletely processed IGF-II binds to IGF-I receptors in the pituitary gland, growth hormone production is suppressed by a feedback inhibition, resulting in serum IGF-I concentration drop.

In our patient the circulating levels of insulin, glucose and IGF-I were low, which distinguishes our case from insulinoma and implies that the mechanism of hypoglycemia was mediated by IGF-II and the forceful insulin effect through the afore-mentioned receptors. Furthermore, the normal IGF-II levels and the elevated ratio of IGF-II/IGF-I of 10:1 (485/28.4 = 17) were indicative of NICTH [17]. Tumor-derived IGF-II plays a crucial role in this type of tumor-related hypoglycemia.

As for most types of cancer, hepatocarcinogenesis is a multi-step process involving different genetic alterations and extraneous factors that ultimately lead to malignant transformation of the hepatocyte. IGF-II is far from being restricted to HCCs but is a frequent phenomenon in many different human malignancies. The growth factor is believed to exert their effect during cellular proliferation through endocrine, autocrine and paracrine mechanisms. Expression of IGF-II in rodents remains high around birth and becomes undetectable within 3 weeks. But the reactivation of this gene has been reported in variety of neoplasms including HCC in rats. IGF-II expression has been shown to be reactivated during progression of neoplastic nodules to HCC and that consists a common phenomenon in hepatocarcinogenesis irrespective of species and the natural history of HCC.

Treatment of HCC-induced hypoglycemia is problematic. The optimal treatment is tumor removal. Partial hepatectomy should be considered in patient without cirrhosis. Orthotopic liver transplantation is the treatment of choice in patients with advanced stage of cirrhosis who fulfill the USNOS criteria [18]. Inoperable cases can be treated with cytoreduction using percutaneous ethanol injection [8], radiofrequency ablation therapy [19], or intrahepatic chemotherapy. Systemic therapies of advanced HCC have not shown favorable outcomes at the present time. Palliative measures to control hypoglycemia apart from definitive therapy include frequent high carbohydrate meals, glucose infusion with or without steroids. Prednisolone is one of the therapeutic options with doses escalating up to 1 mg/kg/day. It counteracts hypoglycemia by stimulating gluconeogenesis. Furthermore, therapeutic modalities include administration of: 1) growth hormone [20, 21] (stimulates multiple growth, anabolic and anti-catabolic effects) in a supra-physiologic dosage can reduce glucose requirement, 2) somatostatin [22] inhibits multiple hormones including growth hormone glucagon, insulin, LH and VIP and generally is ineffective in the treatment of hypoglycemia due to significant decreased levels of glucagon which leads to suppression of hepatic glucose production, 3) diazoxide (not a useful option in NICTH as it works by inhibiting pancreatic insulin release which in these cases is already suppressed) and 4) glucagon infusions.

Glucagon is one of the insulin-regulatory hormones. It stimulates hepatic glucose production by the breakdown of glycogen (glycogenolysis) and by induction of gluconeogenesis. Its action on hepatic glucose output in chronic liver disease patients decreases. Continuous glucagon infusion might be beneficial in hypoglycemia treatment of a non-islet cell tumor. Glucagon stimulation test may be also useful to predict treatment responsiveness and a fast approach that serves to clarify the etiology of hypoglycemia as a diagnostic tool [23]. Nevertheless, although we used most of the methods described above, our patient had no symptomatic response to any of the therapies administered.

In conclusion, tumor hypoglycemia is an uncommon, but potentially lethal disorder. In addition, hypoglycemia deteriorates the patient’s performance status and it is an indicator for worse quality of life. Hypoglycemia disappears with definite treatment of non-islet cell tumors. Resistant hypoglycemia in this specific patient was a result of liver insufficiency and advanced stage hepatoma which were combined with probable IGF-II production in the context of a paraneoplastic syndrome. Finally, the interaction of more than one adverse factor indicates the worse of the prognosis and not only one specific factor such as paraneoplastic phenomenon.
